# A phase 2 trial of neoadjuvant metformin in combination with trastuzumab and chemotherapy in women with early HER2-positive breast cancer: the METTEN study

**DOI:** 10.18632/oncotarget.26286

**Published:** 2018-11-02

**Authors:** Begoña Martin-Castillo, Sonia Pernas, Joan Dorca, Isabel Álvarez, Susana Martínez, Jose Manuel Pérez-Garcia, Norberto Batista-López, César A. Rodríguez-Sánchez, Kepa Amillano, Severina Domínguez, Maria Luque, Agostina Stradella, Idoia Morilla, Gemma Viñas, Javier Cortés, Elisabet Cuyàs, Sara Verdura, Álvaro Fernández-Ochoa, Salvador Fernández-Arroyo, Antonio Segura-Carretero, Jorge Joven, Elsa Pérez, Neus Bosch, Margarita Garcia, Eugeni López-Bonet, Samiha Saidani, Maria Buxó, Javier A. Menendez

**Affiliations:** ^1^ Unit of Clinical Research, Catalan Institute of Oncology, Girona, Spain; ^2^ Department of Medical Oncology, Breast Unit, Catalan Institute of Oncology-Hospital Universitari de Bellvitge-Bellvitge Research Institute (IDIBELL), L'Hospitalet de Llobregat, Barcelona, Spain; ^3^ Medical Oncology, Catalan Institute of Oncology, Girona, Spain; ^4^ Medical Oncology Service, Hospital Universitario Donostia, Donostia-San Sebastián, Spain; ^5^ Biodonostia Health Research Institute, Donostia-San Sebastián, Spain; ^6^ Medical Oncology Department, Hospital de Mataró, Mataró, Barcelona, Spain; ^7^ Baselga Institute of Oncology (IOB), Hospital Quirón, Barcelona, Spain; ^8^ Medical Oncology Service, Hospital Universitario de Canarias, La Laguna, Tenerife, Spain; ^9^ Medical Oncology Service, Hospital Universitario de Salamanca, Salamanca, Spain; ^10^ Instituto de Investigación Biomédica de Salamanca (IBSAL), Salamanca, Spain; ^11^ Medical Oncology, Hospital Universitari Sant Joan, Reus, Spain; ^12^ Medical Oncology Service, Hospital Universitario Araba, Vitoria-Gasteiz, Spain; ^13^ Department of Medical Oncology, Hospital Universitario Central de Asturias, Oviedo, Spain; ^14^ Department of Medical Oncology, Ramón y Cajal University Hospital, Madrid, Spain; ^15^ Metabolism and Cancer Group, Girona Biomedical Research Institute, Girona, Spain; ^16^ Department of Analytical Chemistry, University of Granada, Granada, Spain; ^17^ Research and Development of Functional Food Centre (CIDAF), Health Science Technological Park, Granada, Spain; ^18^ Unitat de Recerca Biomèdica, Hospital Universitari Sant Joan, Institut d'Investigació Sanitària Pere Virgili, Universitat Rovira i Virgili, Reus, Spain; ^19^ Department of Radiology-IDI, Dr. Josep Trueta Hospital of Girona, Girona, Spain; ^20^ Girona Biomedical Research Institute (IDIBGI), Girona, Spain; ^21^ Clinical Research Unit, Catalan Institute of Oncology, L'Hospitalet de Llobregat, Barcelona, Spain; ^22^ Department of Anatomical Pathology, Dr. Josep Trueta Hospital of Girona, Girona, Spain; ^23^ Program Against Cancer Therapeutic Resistance (ProCURE), Metabolism and Cancer Group, Catalan Institute of Oncology, Girona, Spain

**Keywords:** metformin, breast cancer, HER2, trastuzumab

## Abstract

The METTEN study assessed the efficacy, tolerability, and safety of adding metformin to neoadjuvant chemotherapy plus trastuzumab in early HER2-positive breast cancer (BC). Women with primary, non-metastatic HER2-positive BC were randomized (1:1) to receive metformin (850 mg twice-daily) for 24 weeks concurrently with 12 cycles of weekly paclitaxel plus trastuzumab, followed by four cycles of 3-weekly FE75C plus trastuzumab (arm A), or equivalent regimen without metformin (arm B), followed by surgery. Primary endpoint was the rate of pathological complete response (pCR) in the per-protocol efficacy population. pCR rate was numerically higher in the metformin-containing arm A (19 of 29 patients [65.5%, 95% CI: 47.3–80.1]) than in arm B (17 of 29 patients [58.6%, 95% CI: 40.7–74.5]; OR 1.34 [95% CI: 0.46–3.89], *P* = 0.589). The rate of breast-conserving surgery was 79.3% and 58.6% in arm A and B (*P* = 0.089), respectively. Blood metformin concentrations (6.2 μmol/L, 95% CI: 3.6–8.8) were within the therapeutic range. Seventy-six percent of patients completed the metformin-containing regimen; 13% of patients in arm A dropped out because of metformin-related gastrointestinal symptoms. The most common adverse events (AEs) of grade ≥3 were neutropenia in both arms and diarrhea in arm A. None of the serious AEs was deemed to be metformin-related. Addition of anti-diabetic doses of metformin to a complex neoadjuvant regimen was well tolerated and safe. Because the study was underpowered relative to its primary endpoint, the efficacy data should be interpreted with caution.

## INTRODUCTION

Metformin, a biguanide derivative that reduces insulin levels, has long been a cornerstone in the treatment of type 2 diabetes (T2D). There is now compelling evidence to incorporate metformin into the armamentarium against cancer, particularly breast cancer (BC). Notwithstanding the limitations of observational studies, many have consistently indicated that metformin can reduce the incidence, outcome, and mortality of BC in patients with T2D [1–3]. Moreover, preclinical studies have described a variety of molecular mechanisms through which metformin indirectly or directly inhibits the growth of BC cells *in vitro* and *in vivo* [4–6].

The extensive clinical experience accumulated from patients with T2D prescribed metformin, together with its well characterized and modest toxicity profile [7, 8], has significantly shortened the clinical evaluation path of metformin in cancer prevention and treatment [9–11]. Accordingly, many clinical studies, including proof-of-principle studies in the prevention setting and phase 2 trials in the adjuvant and metastatic settings, have been planned and/or are currently under way to test the causal nature of the suggested correlation between metformin and clinical benefit in cancer.

To avoid overestimation of the potential effects of metformin in unselected populations of nondiabetic BC patients, preoperative translational studies are important to define specific BC subgroups more likely to benefit from metformin-based regimens. The neoadjuvant (preoperative) approach is known to maximize the capacity to test the benefits of drug combinations in the context of carefully designed clinical trials of early BC [12–15]. In this regard, a landmark retrospective study revealed that patients with T2D and BC who received metformin and neoadjuvant chemotherapy appeared to have a higher pCR rate than did those not receiving metformin [16], a hypothesis-generating finding that warrants prospective evaluation.

Metformin has been shown to suppress both the tyrosine kinase activity and the expression of the human epidermal growth factor receptor 2 (HER2) protein in *in vitro* models of HER2-overexpressing BC cells [17–20], in addition to prolonging survival in HER2-overexpressing transgenic BC mouse models [21]. Metformin treatment leads also to lower levels of circulating insulin and insulin-like growth factor (IGF-I), and to cell-autonomous inhibition of the mTOR pathway [22–25]. Such a multi-faceted capacity of metformin to target not only HER2 itself but also central mechanisms implicated in refractoriness to HER2-targeted therapies including both the IGF-I/mTOR signaling pathway and the self-renewal/proliferation of tumor-initiating cancer stem cells [26–30] provides strong experimental support to translate these pre-clinical findings into new metformin-based clinical management strategies that may benefit HER2-positive BC patients. However, most of the *in-vitro* models showing anti-HER2 activity of metformin used drug concentrations in the millimolar range, far higher than reported plasma metformin concentrations seen in diabetic patients treated with metformin [27, 31, 32], thereby leaving unanswered the question of whether metformin would have a clinical effect in patients suffering from HER2-positive BC.

The open-label, multicenter, phase II randomized METTEN study [33] (EudraCT number 2011-000490-30) evaluated the clinical activity, tolerability, and safety of adding metformin to neoadjuvant chemotherapy plus trastuzumab in operable, locally advanced, or inflammatory HER2-positive BC.

## RESULTS

### Patient characteristics and disposition

Between June 1, 2012 and March 17, 2016, 98 patients at 10 centers in Spain were recruited into the METTEN study. Due to slow accrual, the study closed prematurely with a reduced sample size after 84 of 244 planned patients were randomly assigned: 41 enrolled patients were allocated to the metformin group (arm A) and 43 patients to the non-metformin group (arm B).

**Figure 1:**
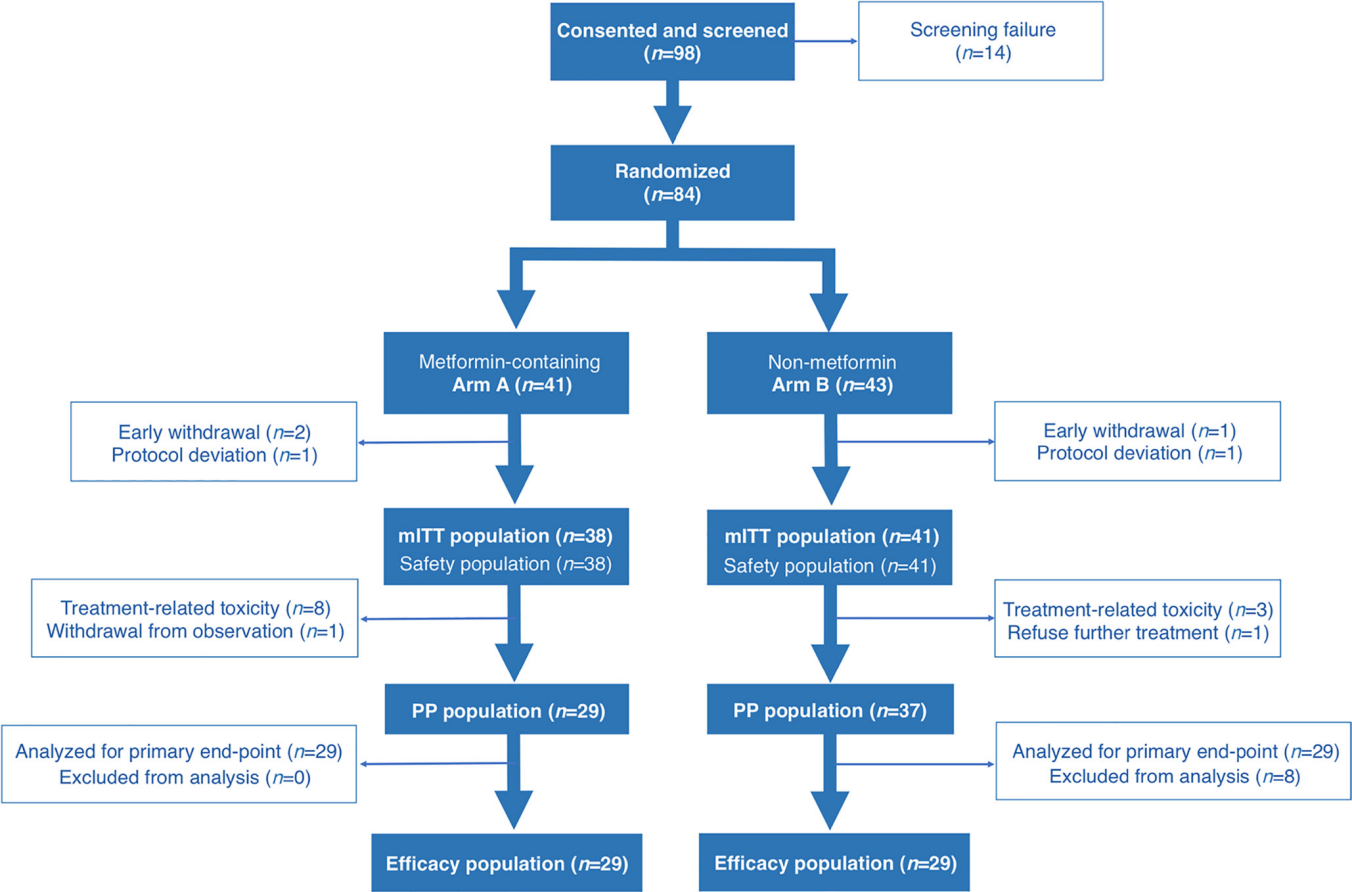
METTEN trial profile CONSORT diagram summarizing disposition of patients in the METTEN study. (mITT: modified intention-to-treat; PP: per-protocol).

**Figure 2:**
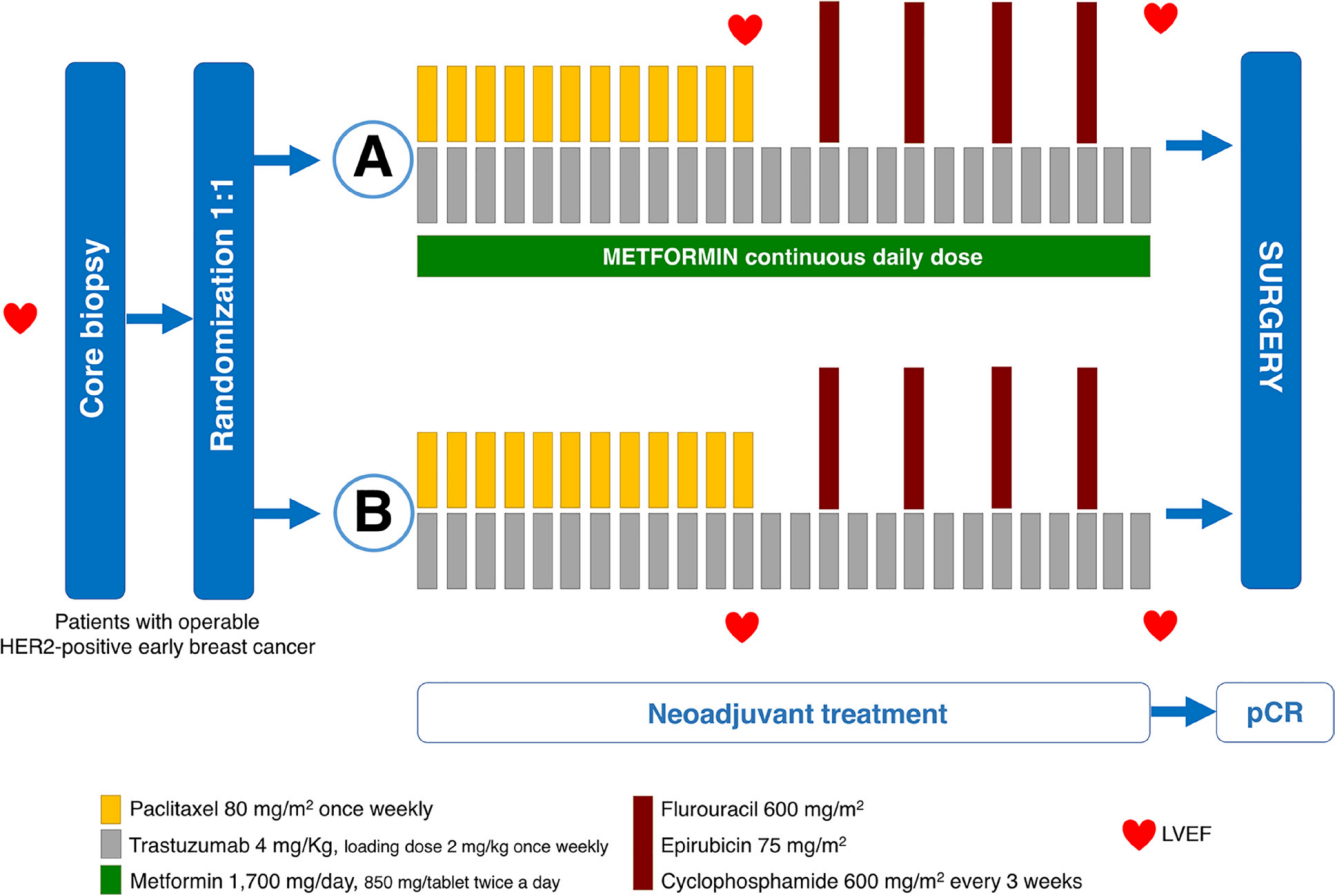
METTEN study design Stratification factors: age, extent of disease (cT2 cN0-1 vs ≥ cT3 or ≥ cN2), and hormone receptor (HR) status. Primary endpoint: pCR in breast and axilla. (HER2, human epidermal growth factor receptor; LVEF, left ventricular ejection fraction; pCR, pathological complete response).

Figure 1 shows the CONSORT diagram summarizing disposition of patients. Fourteen patients did not meet inclusion criteria and were not enrolled at the time of randomization. Nine patients in arm A and four patients in arm B failed to receive their allocated treatment, either due to treatment-related toxicity (eight patients in arm A and three in arm B) or they refused further follow-up or treatment (one in each arm) (Supplementary Table 2). Five patients were excluded from safety analyses because of informed consent withdrawal (two patients in arm A, one patient in arm B) or major protocol violation (one in each arm). The trial profile and treatment schedule is shown in Figure 2.

Patients and tumor characteristics of the modified ITT (mITT) population are summarized in Table 1. The baseline characteristics of the PP population (Supplementary Table 1) were similar to those of the mITT population. Most patients had T2 tumors (66% and 59% in arms A and B, respectively) and lymph node involvement (72% in arms A and B) at diagnosis. Within each stratum, no imbalances in terms of patient characteristics were observed across the two arms. Patients were stratified by age, extent of disease, and HR status.

**Table 1:** Baseline patient demographic and tumor characteristics for the mITT population

	Arm A (*N* = 38)	Arm B (*N* = 41)	*P value*
**Age (years)**			0.780
<50	22 (57.9%)	25 (61.0%)	
≥50	16 (42.1%)	16 (39.0%)	
Mean ± SD (range)	47.2 ± 10.6 (26–75)	48.0 ± 11.5 (23–72)	0.754
**Menopausal status**			0.818
Post	14 (36.8%)	17 (41.5%)	
Pre	24 (63.2%)	24 (58.8%)	
**Body weight (kg)**			
Mean ± SD (range)	64.6 ± 8.7 (45.3–89.0)	65.2 ± 9.4 (48.0–83.0)	0.289
**Body mass index (BMI)**			0.564
<25	21 (55.3%)	20 (48.8%)	
≥25 (overweight)	17 (44.7%)	21 (51.2%)	
**Clinical tumor status**			0.681
cT2	25 (65.8%)	26 (63.4%)	
cT3	12 (31.6%)	10 (24.4%)	
cT4a	0 (0.0%)	1 (2.4%)	
cT4b	1 (2.6%)	3 (7.3%)	
cT4d	0 (0.0%)	1 (2.4%)	
**Clinical nodal Stage**			0.445
cN0	9 (23.7%)	13 (31.7%)	
cN1	24 (63.2%)	20 (48.8%)	
cN2	1 (2.6%)	4 (9.7%)	
cN3	4 (10.5%)	4 (9.7%)	
**Hormone receptor status**			0.477
ER and/or PgR positive	19 (50.0%)	24 (58.5%)	
ER and PR negative	19 (50.0%)	17 (41.5%)	
**Tumor grade**			0.272
G1	2 (7.1%)	0 (0.0%)	
G2	12 (42.9%)	18 (54.5%)	
G3	14 (50.0%)	15 (45.5%)	
Unknown	10	8	
**Baseline LVEF (%)**			0.755
[50–55]	3 (10.3%)	2 (6.3%)	
[55–60]	7 (24.1%)	6 (18.8%)	
[60–65]	8 (27.6%)	13 (40.6%)	
[65–70]	11 (37.9%)	11 (34.4%)	
≥70	9	9	
**Type of programmed surgery**			0.171
Breast-conserving	26 (76.5%)	24 (61.5%)	
Mastectomy	8 (23.5%)	15 (38.5%)	
Unknown	4	2	

LVEF: Left Ventricular Ejection Fraction.

### Responses and surgery

The primary endpoint was the rate of pCR in breast and axilla in the efficacy analyzable PP population (twenty-nine patients in each arm). In arm A, 19/29 PP patients (65.5%, 95% CI: 47.3–80.1%) had a pCR versus 17/29 PP patients (58.6%, 95% CI: 40.7–74.5%) in arm B. The minimum clinically important difference that could be detectable considering the available PP population size (27.7%; α = 0.15, β = 0.20) was included in the upper limit (31.8%) of the confidence interval of the difference of pCR rates between the metformin-containing and the standard reference arm. The combined rates of pCR (ypT0/is, ypN0) and near-pCR, the latter defined as presence of infiltrating residual disease of less than 5 mm and node negativity (ypT1aN0), were 79.3% (95% CI: 61.6–90.2%) in arm A and 72.4% (95% CI: 54.3–85.3%) in arm B (Table 2).

**Table 2:** Surgery and pathologic response in the PP efficacy population

	Arm A (*N* = 29)	Arm B (*N* = 29)	*P* value
**Type of surgery**			
Mastectomy	6 (20.7%)	12 (41.4%)	0.089
Breast-conserving surgery	23 (79.3%)	17 (58.6%)	
**Response**			
pCR			0.588
No	10 (34.5%)	12 (41.4%)	
Yes	19 (65.5%)	17 (58.6%)	
pCR + near pCR			0.539
No	6 (20.7%)	8 (27.6%)	
Yes	23 (79.3%)	21 (72.4%)	
**Type of surgery & response**			
Mastectomy (*N* = 18)			
pCR			0.620
No	4 (66.7%)	5 (41.7%)	
Yes	2 (33.3%)	7 (58.3%)	
pCR + near pCR			0.321
No	4 (66.7%	4 (33.3%)	
Yes	2 (33.3%)	8 (66.7%)	
Breast-conserving surgery (*N* = 40)			
pCR			0.314
No	6 (26.1%)	7 (41.2%)	
Yes	17 (73.9%)	10 (58.8%)	
pCR + near pCR			0.373
No	2 (8.7%)	4 (23.5%)	
Yes	21 (91.3%)	13 (76.5%)	

Breast-conserving surgery (BCS) was possible in 79.3% of patients in arm A, which was apparently superior to the 58.6% achieved in arm B (*P* = 0.089, Table 2). Among patients undergoing BCS, 91.3% achieved a pCR/nearpCR in the metformin arm versus 76.5% in the reference arm (Table 2).

Supplementary Table 3 summarizes the pCR and surgery analyses performed for the mITT population. In Arm A, 19/38 mITT patients (50%, 95% CI: 34.8–65.1%) had a pCR versus 23/41 mITT patients (56.1%, 95% CI: 41.0–70.1%) in arm B. BCS was possible in 78.4% of mITT patients in arm A, which was superior to the 61.0% achieved in arm B (*P* = 0.096).

### Prediction of response

Although the study was underpowered (42%) because of a small number of patients evaluable for the primary endpoint, we performed an exploratory analysis to describe the distribution of pCR rates between arms in the analyzable PP efficacy population. Such exploratory analysis showed no differences between the two arms (odds ratio [OR] 1.34 [95% CI: 0.46–3.89], *P* = 0.589; Table 3). The analysis performed in the mITT population similarly showed no differences between the two arms (OR 0.78 [95% CI: 0.32–1.90], *P* = 0.588; Supplementary Table 4).

**Table 3:** Univariable analysis of factors associated with a pCR in the PP efficacy population

Category	No pCR *N* (*%)*	pCR *N (%)*	OR (95% CI)	*P* value
**Arm**				
B	12 (41.4%)	17 (58.6%)	1	
A	10 (34.5%)	19 (65.5%)	1.34 (0.46–3.89)	0.589
**Age (years)**				
<50	16 (47.1%)	18 (52.9%)		
≥50	6 (25.0%)	18 (75.0%)	2.67 (0.85–8.37)	0.093
**Clinical tumor stage**				
≥T3	12 (52.2%)	11 (47.8%)	1	
T2	10 (28.6%)	25 (71.4%)	3.12 (1.02–9.48)	0.073
**Clinical nodal status**				
*N* ≥ 2	4 (40.0%)	6 (60.0%)	1	
N0–1	18 (37.5%)	30 (62.5%)	1.11 (0.28–4.48)	0.882
**ER**				
Positive	13 (43.3%)	17 (56.7%)	1	
Negative	9 (32.1%)	19 (67.9%)	1.61 (0.55–4.72)	0.381
**PgR**				
Positive	13 (56.5%)	10 (43.5%)	1	
Negative	9 (25.7%)	26 (74.3%)	3.76 (1.23–11.51)	0.021
**HR status**				
Positive	14 (45.2%)	17 (54.8%)	1	
Negative	8 (29.6%)	19 (79.4%)	1.96 (0.66–5.80)	0.227

OR, odds ratio.

In univariable analysis for predetermined factors predicting a pCR in the two arms, solely T2 and PgR negativity (*P* = 0.021) appeared to associate with the probability of achieving pCR (OR 3.12 [95% CI: 1.02–9.48] and 3.76 [95% CI: 1.23–11.51], respectively) in the efficacy analyzable PP population (Table 3). In bivariate analysis, PgR negativity seemed to show predictive capacity irrespective of the arm in which the patients were randomized (Supplementary Table 5). In the mITT population, a similar association appeared to occur between PgR negativity and the probability of achieving pCR in uni- and bivariate analysis (Supplementary Tables 4 and 6, respectively).

In multivariable analysis, PgR negativity no longer associated with the probability of achieving a pCR (data not shown). Supplementary Tables 7 and 8 summarizes how the pCR rates in both arms appeared to remain unchanged according to hormonal receptor status in the PP and mITT populations, respectively.

### Circulating metformin

**Figure 3:**
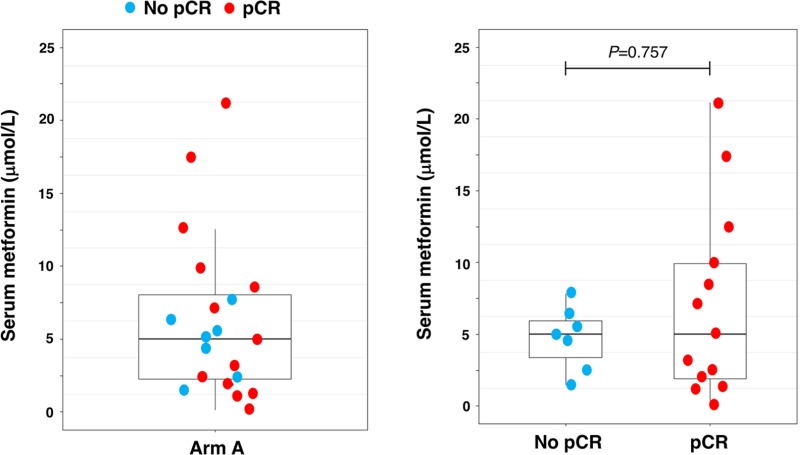
Circulating serum metformin Box plots indicating median (black lines within the boxes), interquartile ranges, whiskers and ranges for post-treatment levels of circulating serum metformin (μmol/L; *N* = 20). (pCR: pathological complete response).

We assessed serum concentrations of metformin in a subgroup of twenty-two patients using HPLC-ESI-QTOF-MS (Figure 3). Inadequate blood samples were drawn in two patients and were excluded from the analysis. The mean concentration was determined to be 6.2 μmol/L (95% CI: 3.6–8.8) with a range from 0.1 μmol/L to 21.1 μmol/L. We detected slightly higher levels of circulating metformin in patients achieving pCR (mean 7.1 μmol/L; 95% CI: 3.0–11.1) than in those belonging to the non-responders group (mean 4.7 μmol/L; 95% CI: 2.7–6.7; *P* = 0.757). Supplementary Figure 1 shows the distribution of serum metformin through concentrations as a function of the time of blood sampling/metformin intake.

### Compliance with treatment and toxicity

The most frequently occurring AEs (290 in arm A and 306 in arm B) were fatigue, diarrhea, nausea, alopecia, sensory neuropathy, mucositis, neutropenia, and elevated AST/ALT (Table 4, Supplementary Table 9). Most AEs were of grades 1 and 2 (92.1% in arm A and 95.8% in arm B; Table 4, Supplementary Table 9). The majority of the most frequent AEs were deemed possibly related to study treatment. The overall incidence of AEs of grade ≥3 ranged from 7.9% (23/290 events) in arm A to 4.3% (13/306 events) in arm B; the most common of which were neutropenia (7/38 patients in arm A and 5/41 patients in arm B) and diarrhea (5 and 0, respectively; Table 4).

**Table 4:** Cardiac and most common adverse events reported as possibly, probably, or definitely related to treatment in the mITT population

	Arm A (*N* = 38)				Arm B (*N* = 41)			
	Grade 1	Grade 2	Grade 3	Grade 4	Grade 1	Grade 2	Grade 3	Grade 4
**Hematological toxicity**								
Anemia	3 (7.9%)	3 (7.9%)	2 (5.3%)	0 (0.0%)	2 (4.9%)	4 (9.8%)	0 (0.0%)	0 (0.0%)
Thrombocytopenia	0 (0.0%)	0 (0.0%)	1 (2.6%)	0 (0.0%)	0 (0.0%)	0 (0.0%)	0 (0.0%)	0 (0.0%)
Leukopenia	3 (7.9%)	0 (0.0%)	0 (0.0%)	0 (0.0%)	3 (7.3%)	0 (0.0%)	0 (0.0%)	0 (0.0%)
Neutropenia	2 (5.3%)	4 (10.5%)	5 (13.2%)	2 (5.3%)	4 (9.8%)	2 (4.9%)	4 (9.8%)	1 (2.4%)
Febrile Neutropenia	0 (0.0%)	0 (0.0%)	1 (2.6%)	1 (2.6%)	0 (0.0%)	0 (0.0%)	2 (4.9%)	0 (0.0%)
**Gastrointestinal disorders**								
Diarrhea	18 (47.4%)	5 (13.2%)	5 (13.2%)	0 (0.0%)	12 (29.3%)	0 (0.0%)	0 (0.0%)	0 (0.0%)
Constipation	4 (10.5%)	0 (0.0%)	0 (0.0%)	0 (0.0%)	2 (4.9%)	1 (2.4%)	0 (0.0%)	0 (0.0%)
Nausea	14 (36.8%)	4 (10.5%)	1 (2.6%)	0 (0.0%)	12 (29.3%)	4 (9.8%)	1 (2.4%)	0 (0.0%)
Vomiting	12 (31.6%)	5 (13.2%)	0 (0.0%)	0 (0.0%)	4 (9.8%)	1 (2.4%)	1 (2.4%)	0 (0.0%)
Mucositis	13 (34.2%)	1 (2.6%)	0 (0.0%)	0 (0.0%)	12 (29.3%)	5 (12.2%)	0 (0.0%)	0 (0.0%)
Dyspepsia	2 (5.3%)	0 (0.0%)	0 (0.0%)	0 (0.0%)	2 (4.9%)	0 (0.0%)	0 (0.0%)	0 (0.0%)
Pyrosis	3 (7.9%)	0 (0.0%)	0 (0.0%)	0 (0.0%)	6 (14.6%)	1 (2.4%)	0 (0.0%)	0 (0.0%)
Epigastric Pain	6 (15.8%)	0 (0.0%)	0 (0.0%)	0 (0.0%)	5 (12.2%)	0 (0.0%)	0 (0.0%)	0 (0.0%)
**General disorders**								
Fatigue	22 (57.9%)	8 (21.1%)	1 (2.6%)	0 (0.0%)	23 (56.1%)	11 (26.8%)	0 (0.0%)	0 (0.0%)
Headache	2 (5.3%)	1 (2.6%)	0 (0.0%)	0 (0.0%)	4 (9.8%)	2 (4.9%)	0 (0.0%)	0 (0.0%)
Fever	1 (2.6%)	0 (0.0%)	0 (0.0%)	0 (0.0%)	2 (4.9%)	0 (0.0%)	0 (0.0%)	0 (0.0%)
**Vascular disorders**								
Edema	2 (5.3%)	0 (0.0%)	0 (0.0%)	0 (0.0%)	4 (9.8%)	0 (0.0%)	0 (0.0%)	0 (0.0%)
Hypertension	0 (0.0%)	0 (0.0%)	0 (0.0%)	0 (0.0%)	2 (4.9%)	0 (0.0%)	0 (0.0%)	0 (0.0%)
**Skin disorders**								
Alopecia	5 (13.2%)	12 (31.6)	0 (0.0%)	0 (0.0%)	9 (22.0%)	9 (22.0%)	0 (0.0%)	0 (0.0%)
Rash	7 (18.4%)	0 (0.0%)	0 (0.0%)	0 (0.0%)	4 (9.8%)	2 (4.9%)	0 (0.0%)	0 (0.0%)
Erythema	2 (5.3%)	0 (0.0%)	0 (0.0%)	0 (0.0%)	4 (9.8%)	0 (0.0%)	1 (2.4%)	0 (0.0%)
Pruritus	4 (10.5%)	0 (0.0%)	0 (0.0%)	0 (0.0%)	2 (4.9%)	0 (0.0%)	0 (0.0%)	0 (0.0%)
Nail changes	3 (7.9%)	2 (5.3%)	0 (0.0%)	0 (0.0%)	4 (9.8%)	2 (4.9%)	0 (0.0%)	0 (0.0%)
Toxicodermic reaction to chemotherapy	0 (0.0%)	0 (0.0%)	0 (0.0%)	0 (0.0%)	0 (0.0%)	0 (0.0%)	1 (2.4%)	0 (0.0%)
Rosacea	0 (0.0%)	0 (0.0%)	0 (0.0%)	0 (0.0%)	0 (0.0%)	0 (0.0%)	1 (2.4%)	0 (0.0%)
Skin toxicity	2 (5.3%)	0 (0.0%)	0 (0.0%)	0 (0.0%)	3 (7.3%)	2 (4.9%)	0 (0.0%)	0 (0.0%)
**Metabolism disorders**								
Anorexia	4 (10.5%)	0 (0.0%)	0 (0.0%)	0 (0.0%)	2 (4.9%)	1 (2.4%)	0 (0.0%)	0 (0.0%)
Hypercalcemia	1 (2.6%)	0 (0.0%)	0 (0.0%)	0 (0.0%)	0 (0.0%)	0 (0.0%)	0 (0.0%)	0 (0.0%)
Hypercholesterolemia	1 (2.6%)	0 (0.0%)	0 (0.0%)	0 (0.0%)	1 (2.4%)	1 (2.4%)	0 (0.0%)	0 (0.0%)
Hypertriglyceridemia	1 (2.6%)	0 (0.0%)	0 (0.0%)	0 (0.0%)	1 (2.4%)	0 (0.0%)	0 (0.0%)	0 (0.0%)
**Metabolism disorders**								
AST/ALT increased	8 (21.1%)	3 (7.9%)	2 (5.3%)	0 (0.0%)	7 (17.1%)	1 (2.4%)	0 (0.0%)	0 (0.0%)
**Musculoskeletal disorders**								
Arthralgia	3 (7.9%)	0 (0.0%)	0 (0.0%)	0 (0.0%)	6 (14.6%)	2 (4.9%)	0 (0.0%)	0 (0.0%)
Myalgia	8 (21.1%)	0 (0.0%)	0 (0.0%)	0 (0.0%)	7 (17.1%)	2 (4.9%)	0 (0.0%)	0 (0.0%)
Septic arthritis	0 (0.0%)	0 (0.0%)	1 (2.6%)	0 (0.0%)	0 (0.0%)	0 (0.0%)	0 (0.0%)	0 (0.0%)
**Nervous system disorders**								
Sensory Neuropathy	10 (26.3%)	1 (2.6%)	0 (0.0%)	0 (0.0%)	19 (46.3%)	5 (12.2%)	0 (0.0%)	0 (0.0%)
Dizziness	1 (2.6%)	1 (2.6%)	0 (0.0%)	0 (0.0%)	1 (2.4%)	1 (2.4%)	0 (0.0%)	0 (0.0%)
Dysgeusia	1 (2.6%)	0 (0.0%)	0 (0.0%)	0 (0.0%)	4 (9.8%)	0 (0.0%)	0 (0.0%)	0 (0.0%)
**Respiratory disorders**								
Epistaxis	3 (7.9%)	0 (0.0%)	0 (0.0%)	0 (0.0%)	9 (22.0%)	0 (0.0%)	0 (0.0%)	0 (0.0%)
Dyspnea	1 (2.6%)	0 (0.0%)	0 (0.0%)	0 (0.0%)	3 (7.3%)	0 (0.0%)	0 (0.0%)	0 (0.0%)
**Reproductive system**								
Amenorrhea	1 (2.6%)	3 (7.9%)	1 (2.6%)	0 (0.0%)	0 (0.0%)	1 (2.4%)	0 (0.0%)	0 (0.0%)
**Cardiac disorders**								
Left ventricular systolic dysfunction	0 (0.0%)	1 (2.6%)	0 (0.0%)	0 (0.0%)	0 (0.0%)	1 (2.4%)	1 (2.4%)	0 (0.0%)
Dilated aortic root	1 (2.6%)	0 (0.0%)	0 (0.0%)	0 (0.0%)	0 (0.0%)	0 (0.0%)	0 (0.0%)	0 (0.0%)

Data are *N* (%).

The number of serious AEs requiring hospitalization was three in arm A and two in arm B (details are summarized in the Supplementary Table 10). No treatment-related deaths occurred.

### Cardiac tolerability

**Figure 4:**
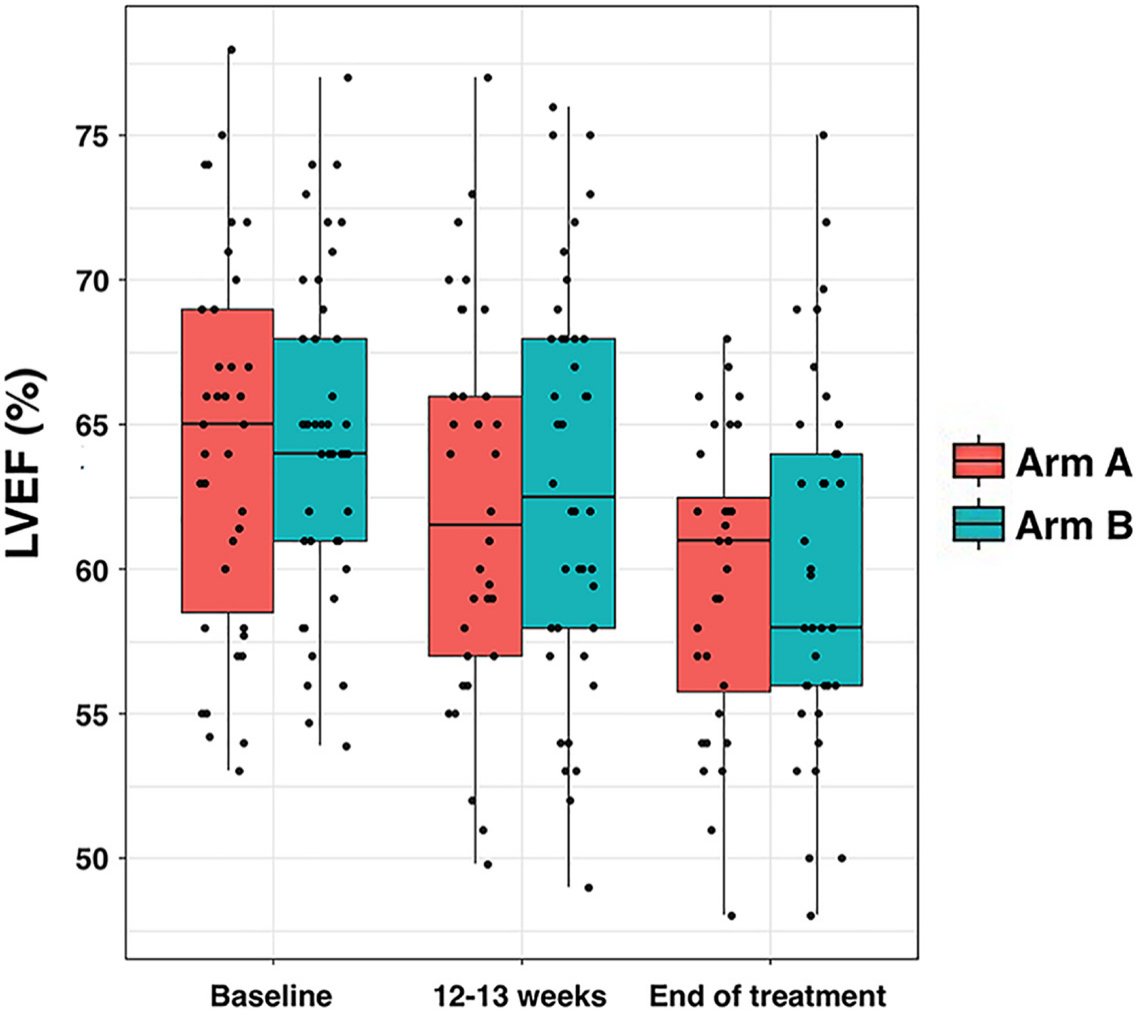
Left ventricular ejection fraction (LVEF) per treatment arm Box plots indicating median (black lines within the boxes), interquartile ranges, whiskers and ranges for LVEF at baseline, after 12–13 weeks, and at the end of therapy.

Table 5 shows baseline LVEF values and changes during neoadjuvant treatment in the two study arms. LVEF dropped below baseline during the treatment period in both arms; however, mean and median decreases were no more than 5% (Supplementary Table 11). Although the profiles of LVEF changes over time were similar between arms (Figure 4), only one (2.9%) patient in arm A and six (15%) in arm B exhibited asymptomatic decreases in LVEF below the institutional lower limit (50%) and >10% from baseline at week 12 (*P* = 0.032; Table 5). At the treatment end, none (0%) of the patients in arm A and three (8.1%) in arm B presented decreases in LVEF (*P* = 0.409; Table 5). Only one patient (2.7%) in arm B experienced symptomatic heart failure.

**Table 5:** Left ventricular ejection fraction (LVEF) in the mITT population

	Arm A	Arm B	
**At baseline**	*N* = 38	*N* = 41	
Median (IQR)	65.0% (58.0 to 69.3)	64.0% (61.0 to 68.5)	
**Week 12**	*N* = 34	*N* = 40	
Median change from baseline (IQR)	–1.5% (–6.6 to 1.2)	–1.0% (–6.8 to 4.0)	
LVEF measurement (*N*, %)			*P* = 0.032
No decrease or decrease <10%, still above LLN	32 (94.1%)	33 (82.5%)	
Decrease <10%, below LLN	1 (2.9%)	1 (2.5%)	
Decrease 10–15%, still above LLN	0 (0.0%)	6 (15.0%)	
Decrease 10–15%, below LLN	0 (0.0%)	0 (0.0%)	
Decrease >15%, still above LLN	1 (2.9%)	0 (0.0%)	
Decrease >15%, below LLN	0 (0.0%)	0 (0.0%)	
**End of treatment**	*N* = 32	*N* = 37	
Median change from baseline (IQR)	–4.0% (–6.0 to –1.8)	–5.0% (–7.5 to –1.0)	
LVEF measurement (*N*, %)			
No decrease or decrease <10%, still above LLN	27 (84.4%)	30 (81.1%)	*P* = 0.409
Decrease <10%, below LLN	1 (3.1%)	0 (0.0%)	
Decrease 10–15%, still above LLN	2 (6.3%)	3 (8.1%)	
Decrease 10–15%, below LLN	0 (0.0%)	3 (8.1%)	
Decrease >15%, still above LLN	2 (6.3%)	1 (2.7%)	
Decrease >15%, below LLN	0 (0.0%)	0 (0.0%)	

Data are median (IQR) or *N* (%) unless stated otherwise.

LLN, lower limit of institutional normal; LVEF, left ventricular ejection fraction.

## DISCUSSION

The METTEN study compared conventional chemotherapy plus trastuzumab with the combination of metformin and chemotherapy plus trastuzumab in the neoadjuvant setting for treatment of early HER2-positive BC.

Assessment of pCR using the definition ypT0/is, ypN0 showed that the two treatment regimens were highly active, with pCR rates ranging from 58.6% in the reference arm to 65.5% in the metformin arm. Such high pCR rates in the small sample size of the METTEN study were consistent with those originally reported in the MD Anderson Cancer Center trial (55–65%) [34, 35] and with our previous experience (61.4%) of concurrent trastuzumab plus weekly paclitaxel-FEC as primary therapy for HER2-positive BC in everyday clinical practice [36]. Although the pCR rates in our study were numerically higher than those generally found in larger randomized phase III trials such as the NOAH [37], the GeparQuattro [38], the HannaH [39], or the Cortazar meta-analysis of neoadjuvant BC trials [13], which reported pCR rates up to 40%, such differences were most likely due to differences in study populations.

The numerically higher pCR rate observed in the PP population receiving the neoadjuvant metformin did not reach statistical significance in our study. However, it should be acknowledged that the trial was closed before the first scheduled interim analysis due to slow recruitment. As a result, a formal statistical comparison of treatment arms in the reported efficacy/PP population was statistically underpowered, and the efficacy analysis should be considered purely exploratory. Evaluation of long-term outcome data such as 5-year DFS together with correlative biological studies evaluating proliferation markers (e.g., Ki-67) and selected predictive factors of response to neoadjuvant treatment in HER2-positive BC (e.g., EGFR and PTEN) are currently underway in our laboratory to adequately appraise whether those patients who received neoadjuvant metformin might gain an additional survival benefit and the mechanisms involved [35, 40–44]. Although a higher BCS rate was observed in patients receiving additional metformin, breast conservation is known to depend on multiple parameters including breast size, tumor location, presence of DCIS, the multifocality of the lesion, or patient willingness [45], thus making it challenging to attribute such differences to a true clinico-molecular benefit in those patients receiving neoadjuvant metformin.

Data from the Asian Medical Center Breast Cancer Database concluded that diabetic patients receiving metformin when BC is diagnosed show a better prognosis only if they had HR-positive, HER2-positive tumors [46]. Moreover, an analysis of the ALTTO Phase III randomized trial, which assigned patients with HER2-positive BC to receive 1 year of trastuzumab alone, lapatinib alone, their sequence, or their combination, found that metformin exerted a statistically-significant beneficial effect in those patients with diabetes who had primary HER2-positive and HR-positive BC [47]. Neoadjuvant trials with anti-HER2 therapy have demonstrated a HR status-related prognostic value after achieving a pCR, with a higher survival effect in the HR-negative group than in the HR-positive group [48]. In the METTEN study, a higher percentage of PgR-negative patients achieved a pCR in both arms, thus confirming the notion that the likelihood of response according to HR status is an intrinsic characteristic of HER2-positive tumors [49, 50]. However, we failed to clarify the actual predictive value of pCR in the metformin-containing arm according to HR status.

After hepatic uptake, the plasma concentration of metformin is reduced to 5–20 μmol/L after oral doses of 0.5–1.5 g metformin in humans with a mean plasma half-life of about 20 h [6, 31, 51]. Our analytical determination of serum metformin confirmed that treatment of non-diabetic HER2+ BC patients with oral metformin (850 mg twice-daily) for 24 weeks produced blood levels of circulating metformin (approx. 7 μmol/L) equivalent to those generally achieved in diabetic patients at the usual clinical doses and schedules [27, 31, 32]. Although systemic exposure of metformin seemed more elevated in those patients achieving a pCR than in non-responder patients, two outliers within the responder group appeared to drive such trend that failed to reach statistical significance. Moreover, we measured circulating concentrations of metformin in blood samples that were not strictly timed in terms of hours since preceding oral dose [51] and, therefore, our data need to be viewed cautiously in terms of any association between achieved serum concentration and probability of pCR.

One major concern regarding the utility of metformin is its known ability to induce gastrointestinal upset and diarrhea, which might limit patient compliance, particularly when combined with cytotoxic chemotherapy [52]. The METTEN study confirms that metformin is likely a tolerable and safe addition to current therapy regimens [53, 54]. From the perspective of tolerability, it should be noted that the dropout rate in the metformin arm was much lower than the expected 25%; only 13% (5 out of 38) of patients withdrew because of metformin-related gastrointestinal upset and diarrhea, whereas more than 75% (29 out of 38) patients completed the 6-month intervention with metformin as part of a complex neoadjuvant combination. The safety of the triple regimen of metformin, chemotherapy, and trastuzumab was similar to that of chemotherapy and trastuzumab. None of the three serious AEs in arm A was deemed to be exclusively metformin-related. Because residual disease after neoadjuvant therapy is a poor prognostic factor [55], it would be relevant to evaluate whether upregulated mitochondrial oxidative phosphorylation (OXPHOS) –a primary target of metformin- is part of the metabolic shifts that drive tumor recurrence in residual BC [56], thereby allowing metformin to be considered as a safe candidate to treat OXPHOS-dependent residual BC disease.

When we evaluated the cardiac tolerability of metformin given in the triple regimen, metformin did not increase the baseline rate of cardiac dysfunction observed in the reference arm. Moreover, by assessing the trajectories of LVEF decline over time, we observed a small trend towards a lower number of asymptomatic cardiac events in the metformin-containing arm. HER2 signaling is involved in myocardial homeostasis and its inhibition may explain the increased incidence of cardiomyopathy associated with the treatment with trastuzumab, particularly in those patients exposed to cardiotoxic chemotherapies such as anthracyclines [57, 58]. Trastuzumab-induced cardiomyopathy relates, at least in part, to its inability to activate pro-survival catabolic pathways through AMP-activated protein kinase (AMPK) in cardiac cells [59, 60]. Because metformin treatment has been shown to improve cardiovascular function and reduce cardiovascular risk in diabetic patients through the activation of AMPK [61]–a cell-autonomous mechanism that also underlies the activity of metformin as an anticancer drug [4–6]– larger and longer-term studies evaluating biomarkers of cardiotoxicity in trastuzumab-exposed oncologic populations will be needed to clarify whether metformin induces AMPK (and downstream catabolic) signaling upon trastuzumabinduced metabolic dysregulation in cardiomyocytes [20, 62].

During recruitment, the findings of the phase II NeoSphere [49] and TRYPHAENA trials [63], together with the impressive survival benefits for women with HER2-positive metastatic BC receiving pertuzumab along with trastuzumab in the phase III CLEOPATRA study [64], led to the accelerated approval of pertuzumab by the FDA in September 2013 and the European Medicines Agency in July 2015 for use in combination with trastuzumab plus chemotherapy for neoadjuvant treatment of patients with HER2-positive locally advanced, inflammatory, or early-stage BC. Consequently, ethical issues arose during the METTEN study trial based on the recommended standard of care supported by national and international guidelines with a neoadjuvant combination of taxane-containing chemotherapy and a dual blockade of trastuzumab and pertuzumab. Moreover, we cannot exclude the possibility that a rejection bias might exist against the repurposing of generic non-cancer metformin as oncological treatment when confronted to commercially developed anti-cancer drugs [65].

Two previous randomized phase II trials have shown that metformin in combination with systemic therapy fails to significantly improve outcomes in patients with advanced/metastatic pancreatic cancer [66, 67]. These studies by Kordes [66] and Reni [67] intended very ambitious clinical targets in terms of overall survival (from 50% to 75% at 6 months) and progression-free survival (from 50% to 70% at 6 months), respectively. Because the METTEN trial failed to identify also a large difference, i.e., a 25% increase over an expected pCR of 60% with chemotherapy plus trastuzumab before a phase 3 trial could be justified, it might be tempting to suggest that testing against high bars of clinical outcome endpoints instead of using *a priori* non-inferiority trial designs should be cautiously considered before concluding that studies using metformin for treating cancer should be abandoned. Moreover, negative results of first-generation cancer trials using metformin at the same dose and route of administration that in diabetic patients would not rule out the clinical utility of biguanides other than metformin (e.g., phenformin) or non-conventional routes for administering biguanides if previously optimized for oncology indications [68–71]. However, as we did not achieve the target number of patients to power the study, we cannot be certain whether the lack of significant difference between the two arms of the METTEN trial is a type II error or reflects a true lack of efficacy for the metformin-based neoadjuvant strategy in early HER2-positive BC. Beyond general considerations such as the need to consolidate prognostic, predictive, and pharmacodynamic factors of the metabolic response to metformin for selecting subsets of patients most likely to benefit from metformin treatment, mature results from large, randomized studies, such as the NCIC CTG MA.32, the most advanced adjuvant trial investigating the effects of metformin *versus* placebo on invasive DFS and other outcomes on early BC in 3,649 women [10], will be of great interest to confirm or reject [72] the causal nature of the suggested correlation between metformin use and survival benefit in BC patients.

## MATERIALS AND METHODS

### Study design and objectives

Patients were randomly assigned to receive daily metformin (850 mg twice-daily) for 24 weeks concurrently with 12 cycles of weekly paclitaxel plus trastuzumab followed by four cycles of 3-weekly fluorouracil, epirubicin, cyclophosphamide plus trastuzumab (arm A) or equivalent sequential chemotherapy plus trastuzumab without metformin (arm B), followed by surgery.

The primary end point was pCR, defined as absence of invasive tumor cells on hematoxylin and eosin evaluation of the complete resected breast specimen (and all sample regional lymph nodes if lymphadenectomy was performed) following the completion of neoadjuvant systemic therapy. Residual ductal carcinoma *in situ* (DCIS) only was included in the definition of pCR (ypT0/is, ypN0). Secondary aims included the tolerability and safety profile of the metformin-based neoadjuvant combination including cardiac toxicity, the rate of breast conservation, 5-year disease-free survival (DFS), the inhibition of tumor tissue biomarkers (including proliferative, mTOR/AMPK- and HER2-related pathways), and changes in circulating levels of insulin and metabolites. Studies of disease free survival rates and correlative biological markers are ongoing and will be reported separately. Independent institutional review boards approved the study protocol and any amendments. Written informed consent was obtained from each participant. The study was registered with the EU Clinical Trials Register and is available online (https://www.clinicaltrialsregister.eu/ctr-search/trial/2011-000490-30/ES).

### Patient selection

Patients were eligible if they met the following criteria: previously untreated, operable, locally advanced, or inflammatory BC >2.0 cm in largest clinical diameter, and confirmed HER2 positivity (either immunohistochemistry 3+ or 2+ and positive for fluorescent or chromogenic *in situ* hybridization). Patients were excluded from this study if they had impaired cardiac function (e.g., uncontrolled or symptomatic angina, clinically significant arrhythmias, congestive heart failure, transmural myocardial infarction), uncontrolled hypertension, concurrent treatment with therapies that can alter insulin levels (including chronic treatment with oral corticoids), metabolic disease (e.g., diabetes mellitus type I or II, obesity [BMI >30], impaired glucose tolerance [>128 mg/dL], hypercholesterolemia or hypertriglyceridemia of grade ≥3 according to CTC-NCIC version 4.0). See Supplementary Materials for additional inclusion and exclusion criteria.

### Treatment

Chemotherapy consisted of weekly paclitaxel (80 mg/m^2^) for 12 weeks, concomitant trastuzumab (4 mg/kg loading dose followed by 2 mg/kg weekly for 12 weeks), followed by four courses of fluorouracil (600 mg/m^2^), epirubicin (75 mg/m^2^), and cyclophosphamide (600 mg/m^2^) (FE_75_C), administered every 3 weeks with concomitant trastuzumab (6 mg/kg). Corticosteroids and histamine-receptor blockers were administered before paclitaxel. Patients on arm A received concomitant metformin (850 mg twice-daily) for 24 weeks, which was given in divided doses with meals, with gradual dose escalation to reduce gastrointestinal side effects. The starting dose was 425 mg (one-half of a tablet) daily with dinner; dosage increase was carried out in increments of 425 mg every week to a total of 850 mg twice-daily after 4 weeks. Patients had surgery within 4–5 weeks of the last cycle of neoadjuvant treatment. Post-surgery, patients received 3-weekly trastuzumab to complete 1 year of neoadjuvant-adjuvant therapy. Radiotherapy and endocrine therapy were according to local guidelines.

### Randomization and masking

Patients were randomly assigned (1:1 ratio) to arm A or arm B with a dynamic randomized block design and a minimization technique. Stratification factors were: age (<50 years vs ≥50 years); clinical tumor size (T2 [2–5 cm diameter] vs ≥T3 [>5 cm diameter]); clinical involvement of axillary lymph nodes (N0-1 vs ≥2); and hormone receptor (HR) status (estrogen receptor or progesterone-receptor [PgR] positive; or both, *vs* estrogen-receptor and PgR negative). Two hundred and fifty-six randomization codes were generated with a block size of 16 patients (8 per arm) per combined strata. On verification of patients' eligibility, investigators were immediately notified of the allocated treatment.

### Assessments

Grading of all adverse events (AEs) was made using National Cancer Institute Common Terminology Criteria for Adverse Events (NCI CTCAE) version 4.0, and reported as cumulative incidence. Cardiac safety was monitored *via* incidence of significant asymptomatic left ventricular systolic dysfunction (LVSD), which was defined as ≥10% decline in left ventricular ejection fraction (LVEF) from baseline to <50% over the course of neoadjuvant treatment. LVEF was evaluated before study, after 12 weeks, and at the completion of treatment. Symptomatic LVSD was reported as a serious AE. Ink marks or surgical clips were used to mark the tumor bed before beginning neoadjuvant therapy, to facilitate surgical procedures and pathology. The Oracle Clinical^®^ software tool was employed to assist with data management, data entry, and data validation.

### Analytical determination of circulating metformin in serum

Serum was collected at the end of the 24-week intervention and stored at −80°C until assayed. Metformin concentrations were determined using high-performance liquid chromatography coupled to electrospray ionization and quadrupole time-of-flight mass spectrometry (HPLC-ESI-QTOF-MS). See Supplementary Materials for a detailed description of the analytical method.

### Sample size and statistical analysis

A Jung's two-stage design for randomized phase II trials with a prospective control [73] was used to estimate the sample size. To keep the sample size small and the study period short, we employed a relatively large type I error (α = 15%) and a short-term outcome variable, the percentage of pCR as primary endpoint, which allowed for early termination of the study if the metformin containing arm failed to show efficacy at the interim analysis. The combination metformin plus chemotherapy/trastuzumab was considered worthwhile if a pCR ≥75% was obtained. By setting an α level of 0.15, a power of 0.80, a balanced allocation (1:1), and an expected drop-out rate of 25%, the sample size was 47 patients for arms A and B to ensure a per-protocol (PP) assessment of pCR in 37 patients in each arm at the first stage. Only if at least two more patients achieved a pCR in the metformin-containing arm than in the reference arm, and provided no safety issues were identified, would the clinical trial proceed to the second stage. In such case, an additional recruitment of 65 patients for arms A and B (to ensure a PP assessment of pCR in 52 patients in each arm), will proceed. The metformin-containing arm was considered effective if 5 or more additional patients achieved a pCR in comparison with the reference arm at the end of the study (*N* = 224 patients at the planned final sample size).

The modified intention-to-treat (mITT)/safety population included all randomly assigned patients who received at least one dose of study medication. The PP/efficacy population included all participants in the mITT population who had not violated any inclusion or exclusion criteria or deviated from the protocol in a way that could affect their efficacy assessments including sufficient treatment duration.

Safety and efficacy parameters were evaluated descriptively. Categorical parameters are presented as frequencies (*N*, %) and were compared using a chi-squared test (or Fisher's exact test, when appropriate). Continuous variables are presented as mean ± standard deviation or median (1st/3rd quartile) and were compared using Student's unpaired *t*-test or the Mann–Whitney *U* test when data were not normally distributed. Data normality before statistical analyses was assessed with the Kolmogorov-Smirnov test. Binary logistic regression was used to assess the prognostic effect of baseline characteristics on pCR. Unadjusted and adjusted odds ratios (ORs) with their relative 95% confidence intervals (CIs) were reported as a measure of association. After 84 of 224 planned patients were randomized, the trial was closed early due to slow recruitment, which left the study underpowered relative to its primary endpoint (*i.e*., pCR). Therefore, the analyses presented here are considered exploratory and *P* values should not be used for drawing conclusions about the impact on pCR when adding neoadjuvant metformin to trastuzumab and chemotherapy.

Statistical analyses were carried out using SPSS (IBM Corp. released 2016. IBM SPSS Statistics for Windows, Version 24.0; Armonk, NY) and STATA (StataCorp. 2013. Stata Statistical Software: Release 13; StataCorp LP, College Station, TX).

## CONCLUSIONS

Larger studies are needed to determine if the similar high percentages of pCR observed in both treatment arms in the METTEN study reflects true lack of clinical efficacy of metformin or whether the study was underpowered for drawing conclusions about metformin effectiveness. Nevertheless, the METTEN study provides useful information, revealing that the addition of a conventional anti-diabetic dose of metformin to complex neoadjuvant regimens involving anthracycline/taxane-based chemotherapy and targeted therapies such as trastuzumab is well tolerated and safe.

## SUPPLEMENTARY MATERIALS FIGURE AND TABLES



## Abbreviations

METTEN: Metformin and Trastuzumab in Neoadjuvancy (METformina y Trastuzumab En Neoadyuvancia Spanish)
BC: Breast Cancer
pCR: pathological Complete Response
AEs: Adverse effects
FE75C: Fluorouracil, Epirubicin (75 mg/m^2^)
T2D: Type 2 diabetes
HR: Hormone Receptor
ER: Estrogen Receptor
PgR: Progesterone receptor
LEVF: Left Ventricular Ejection Fraction
PP: Per-Protocol
ITT: Intention-to-treat
HPLC-ESI-QTOF-MS: High-performance liquid chromatography coupled to electrospray ionization and quadrupole time-of-flight mass spectrometry
OXPHOS: Oxidative Phosphorylation
ORs: Odds Ratios
