# Supplementary material for: A phase 2 trial of neoadjuvant metformin in combination with trastuzumab and chemotherapy in women with early HER2-positive breast cancer: the METTEN study
Source: Oncotarget. 2018 Nov 2;9(86):35687–704. doi: 10.18632/oncotarget.26286 (PMC6235018; doi:10.18632/oncotarget.26286)
Supplement: Supplementary file 1 [file oncotarget-09-35687-s001.pdf]

A phase 2 trial of neoadjuvant metformin in combination with trastuzumab and chemotherapy in women with early HER2-positive breast cancer: the METTEN study

SUPPLEMENTARY MATERIALS

MATERIALS AND METHODS

Inclusion criteria

Age 18–75 years, baseline Eastern Cooperative Oncology Group performance status 0 or 1, and baseline left ventricular ejection fraction (LVEF) ≥50% measured by echocardiography or multiple gated acquisition, normal organ and bone marrow function (absolute neutrophil count ≥1,500/µL, platelets ≥100,000/µL, total bilirubin ≤1.5 times the upper limit of normal [ULN], serum creatinine ≤1.5 times the ULN, AST and ALT ≤2.5 times the ULN), ability to swallow and retain oral medication, and blood glucose levels ≥70 mg/dL (3.9 mmol/L).

Exclusion criteria

Key exclusion criteria were metastatic disease, bilateral BC, any prior treatment for BC, other malignancies or less than 10 years from prior malignancies (except curatively treated basal cell carcinoma, squamous cell carcinoma of the skin, or carcinoma *in situ* of the cervix), inadequate renal function (creatinine clearance <60 mL/min), impaired liver function, enolism (average consumption of 3 alcoholic beverages/day), significant dementia, altered mental status (or any psychiatric condition that would prohibit the understanding or rendering of informed consent), pregnancy and lactation.

Analytical method for determination of metformin in serum

Serum samples were thawed on ice, spiked with 5 ppm of phenformin as internal standard, and treated with methanol to remove protein content before analysis. The mixture was vortex-assisted and then centrifuged for 10 min at 10,000 r.p.m. at 4° C in a Sorvall ST 16R Centrifuge (Thermo Scientific, Waltham, MA, USA). The supernatants were then analyzed by HPLC-ESI-QTOF-MS using an Agilent 1260 HPLC instrument (Agilent Technologies, Palo Alto, CA, USA) coupled to an Agilent 6540 Ultra High Definition (UHD) Accurate Mass Q-TOF equipped with a Jet Stream dual ESI interface. Compounds were separated using a reversed-phase C18 analytical column (Agilent Zorbax Eclipse Plus, 1.8 µm, 4.5 × 150 mm) protected by a guard column cartridge containing the same packing material as the analytical column. The mobile phases were an aqueous solution of ammonium acetate (5 mmol/L, pH=4) and acetonitrile as solvent A and B, respectively. The following gradient of the mobile phases was used to obtain an efficient separation: 0.0–5.0 min [A:B 95/5], 25.0 min [A:B 80/20], 30.0 min [A:B 5/95], 35.0–45.0 min [A:B 95/5]. The column and auto-sampler compartment temperatures were set at 25° C and 4° C, respectively, whereas the follow rate and the injection volume were 0.5 mL/min and 5 µL. The detection was performed in positive-ion mode at a range of 100–500 *m/z*. Ultrahigh pure nitrogen was used as both, drying and nebulizing gas at temperatures of 200° C and 325° C and flows of 10 and 12 L/min, respectively. The MS data were processed using MassHunter workstation software (version B.07.00, Qualitative Analysis (Agilent Technologies, USA).

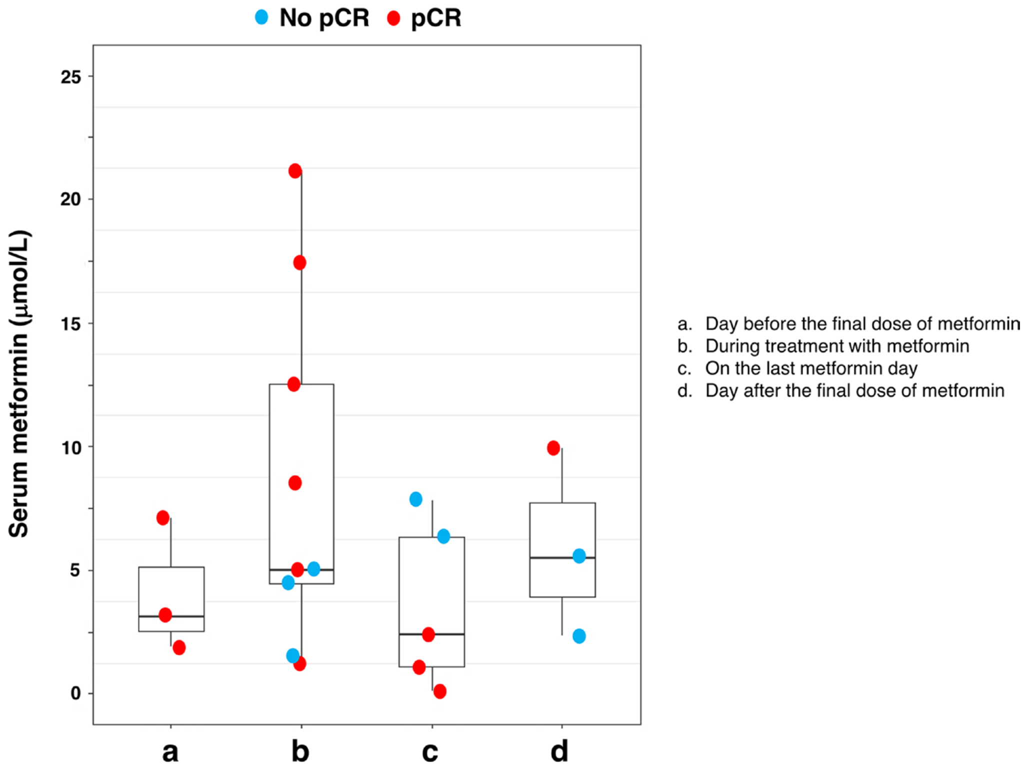

Supplementary Figure 1: Circulating serum metformin. Box plots indicating median (black lines within the boxes), interquartile ranges, whiskers and ranges for post-treatment levels of circulating serum metformin (μmol/L). (pCR: pathological complete response).

Supplementary Table 1: Baseline patient demographic and tumor characteristics for the PP efficacy population

	Arm A (*N* = 29)	Arm B (*N* = 29)	*P* value
**Age (years)**			0.594
<50	18 (62.1%)	16 (55.2%)	
≥50	11 (37.9%)	13 (44.8%)	
Mean ± SD (range)	45.3 ± 9.8 (26–65)	48.5 ± 11.8 (23–71)	0.254
**Menopausal status**			0.286
Post	10 (34.5%)	14 (48.3%)	
Pre	19 (65.5%)	15 (51.7%)	
**Body weight (kg)**			
Mean ± SD (range)	64.7 ± 9.8 (45.3–89.0)	64.2 ± 9.0 (48.0–82.0)	0.634
**Body-mass index (BMI)**			0.793
<25	15 (51.7%)	14 (48.3%)	
≥25 (overweight)	14 (48.3%)	15 (51.7%)	
**Clinical tumor status**			0.300
cT2	19 (65.5%)	17 (58.6%)	
cT3	10 (34.5%)	9 (31.0%)	
cT4a	0 (0.0%)	1 (3.4%)	
cT4b	0 (0.0%)	2 (6.9%)	
**Clinical nodal stage**			0.652
cN0	8 (27.6%)	8 (27.6%)	
cN1	17 (58.6%)	15 (51.7%)	
cN2	1 (3.4%)	4 (13.7%)	
cN3	3 (10.3%)	2 (6.9%)	
**Hormone receptor status**			0.188
ER & PgR			
ER and/or PgR positive	13 (44.8%)	18 (62.1%)	
ER and PgR negative	16 (55.2%)	11 (37.9%)	
**Tumor grade**			0.197
G1	2 (10.0%)	0 (0.0%)	
G2	7 (35.0%)	14 (58.3%)	
G3	11 (55.5%)	10 (41.7%)	
Unknown	9	5	
**Baseline LVEF (%)**			0.499
(50–55)	3 (10.3%)	1 (3.4%)	
(55–60)	7 (24.1%)	5 (17.2%)	
(60–65)	6 (20.7%)	11 (37.9%)	
(65–70)	9 (31.0%)	9 (31.0%)	
≥70	4 (13.8%)	3 (10.3%)	
**Type of programmed surgery**			0.107
Breast-conserving	21 (80.8%)	18 (64.3%)	
Mastectomy	5 (19.2%)	10 (35.7%)	
Unknown	3	1	

Supplementary Table 2: Causes for treatment interruption in the mITT population

Patient code	Treatment Arm	Reason	Related to treatment
619	A	Tzb was interrupted (week 22) due to reduced LVEF (grade 2)	Definite (trastuzumab)
1210	A	Patient declines further follow-up evaluation	Unrelated
1501	A	Diarrhea (grade 3)	Definite (metformin), probable (paclitaxel)
1508	A	Diarrhea (grade 3)	Definite (metformin), probable (paclitaxel)
1509	A	Diarrhea (grade 3)	Definite (metformin), probable (paclitaxel)
1510	A	Inter-recurrent disease (septic arthritis, grade 3)	Possible (metformin, FEC)
1523	A	Diarrhea (grade 3)	Definite (metformin), probable (paclitaxel)
1525	A	Diarrhea (grade 3)	Definite (metformin), probable (paclitaxel)
1526	A	Allergic reaction (grade 2)	Probable (metformin)
603	B	Reduced LVEF (grade 2)	Definite (trastuzumab)
607	B	Skin toxicity (grade 2); Reduced LVEF (grade 2)	Definite (paclitaxel, trastuzumab)
1007	B	Patient refuse further treatment (4th FEC-Tzb cycle)	Unrelated
1518	B	Symptomatic LVEF decline (grade 3)	Definite (trastuzumab)

Tzb: Trastuzumab.

Supplementary Table 3: Surgery and pathologic response in the mITT population

	Arm A (*N* = 38)	Arm B (*N* = 41)	*P* value
**Type of surgery**			
Mastectomy	8 (21.6%)	16 (39.0%)	0.096
Breast-conserving surgery	29 (78.4%)	25 (61.0%)	
**Response**			
pCR			0.587
No	19 (50.0%)	18 (43.9%)	
Yes	19 (50.0%)	23 (56.1%)	
**Type of surgery & response**			
Mastectomy (*N* = 24)			
pCR			0.211
No	6 (75.0%)	7 (43.8%)	
Yes	2 (25.0%)	9 (56.3%)	
Breast-conserving surgery (*N* = 54)			
pCR			0.846
No	12 (41.4%)	11 (44.0%)	
Yes	17 (58.6%)	14 (56.0%)	

Supplementary Table 4: Univariable analysis of factors associated with a pCR in the mITT population

Category	No pCR *N* (%)	pCR *N* (%)	OR (95% CI)	*P value*
**Arm**				
B	18 (43.9%)	23 (56.1%)	1	
A	19 (50.0%)	19 (50.0%)	0.78 (0.32–1.90)	0.588
**Age (years)**				
<50	24 (51.1%)	23 (48.9%)		
≥50	13 (40.6%)	19 (59.4%)	1.53 (0.62–3.78)	0.362
**Clinical tumor stage**				
≥T3	15 (53.6%)	13 (46.4%)	1	
T2	22 (43.1%)	29 (56.9%)	1.52 (0.60–3.84)	0.375
**Clinical nodal status**				
$N \geq 2$	6 (46.2%)	7 (53.8%)	1	
N0–1	31 (47.0%)	35 (53.0%)	0.97 (0.29–3.19)	0.957
**ER**				
Positive	22 (52.4%)	20 (47.6%)	1	
Negative	15 (40.5%)	22 (59.5%)	1.61 (0.66–3.94)	0.294
**PgR**				
Positive	20 (64.5%)	11 (35.5%)	1	
Negative	17 (35.4%)	31 (64.6%)	3.32 (1.29–8.52)	0.013
**HR status**				
Positive	23 (53.5%)	20 (46.5%)	1	
Negative	14 (38.9%)	22 (61.1%)	1.81 (0.74–4.44)	0.197

OR, odds ratio.

Supplementary Table 5: Bivariable analysis of factors associated with pCR in the PP efficacy population

Category		*N*	OR (95% CI)	*P* value
**Arm**				
	B	29	1	
	A	29	1.46 (0.49–4.39)	0.498
**Age (years)**				
	<50	34	1	
	≥50	24	2.76 (0.87–8.67)	0.085
**Arm**				
	B	29	1	
	A	29	1.26 (0.42–3.81)	0.680
**Clinical tumor stage**				
	≥T3	23	1	
	T2	35	2.67 (0.89–8.07)	0.081
**Arm**				
	B	29	1	
	A	29	1.33 (0.46–3.89)	0.596
**Clinical nodal status**				
	$N \geq 2$	10	1	
	N0-1	48	1.07 (0.26–4.36)	0.921
**Arm**				
	B	24	1	
	A	20	1.40 (0.40–4.86)	0.596
**Tumor grade**				
	G3	21	1	
	G1/2	23	1.47 (0.43–5.07)	0.538
**Arm**				
	B	29	1	
	A	29	1.26 (0.43–3.72)	0.670
**ER**				
	Positive	30	1	
	Negative	28	1.57 (0.53–4.62)	0.417
**Arm**				
	B	29	1	
	A	29	1.32 (0.43–4.03)	0.630
**PgR**				
	Positive	23	1	
	Negative	35	3.74 (1.22–11.49)	0.021
**Arm**				
	B	29	1	
	A	29	1.21 (0.41–3.59)	0.734
**HR status**				
	Positive	31	1	
	Negative	27	1.90 (0.63–5.71)	0.256

OR, odds ratio.

Supplementary Table 6: Bivariable analysis of factors associated with pCR in the mITT population

Category		*N*	OR (95% CI)	*P* value
**Arm**				
	B	41	1	
	A	38	0.77 (0.32–1.88)	0.566
**Age (years)**				
	<50	47	1	
	≥50	32	1.54 (0.62–3.83)	0.352
**Arm**				
	B	41	1	
	A	38	0.77 (0.32–1.88)	0.570
**Clinical tumor stage**				
	≥T3	28	1	
	T2	51	1.53 (0.61–3.88)	0.367
**Arm**				
	B	41	1	
	A	38	0.78 (0.32–1.90)	0.589
**Clinical nodal status**				
	$N \geq 2$	13	1	
	N0–1	66	1.00 (0.30–3.30)	0.994
**Arm**				
	B	41	1	
	A	38	0.63 (0.23–1.75)	0.375
**Tumor grade**				
	G3	29	1	
	G1/2	32	0.79 (0.29–2.19)	0.655
**Arm**				
	B	41	1	
	A	38	0.76 (0.31–1.85)	0.541
**ER**				
	Positive	42	1	
	Negative	37	1.65 (0.67–4.04)	0.277
**Arm**				
	B	41	1	
	A	38	0.82 (0.3–2.07)	0.676
**PgR**				
	Positive	31	1	
	Negative	48	3.29 (1.28–8.46)	0.014
**Arm**				
	B	41	1	
	A	38	0.74 (0.30–1.82)	0.508
**HR status**				
	Positive	43	1	
	Negative	36	1.86 (0.75–4.61)	0.179

OR, odds ratio.

Supplementary Table 7: Association of hormonal receptor status with a pCR in the PP efficacy population

Sub-population	No pCR *N (%)*	pCR *N (%)*	OR (95% CI)	*P value*
**ER positive**				
Arm B	7 (41.2%)	10 (58.8%)	1	
Arm A	6 (46.2%)	7 (53.2%)	0.82 (0.19–3.50)	0.785
**ER negative**				
Arm B	5 (41.7%)	7 (58.3%)		
Arm A	4 (25.0%)	12 (75.0%)	2.14 (0.43–10.74)	0.354
**PgR positive**				
Arm B	7 (58.3%)	5 (41.7%)	1	
Arm A	6 (54.6%)	5 (45.4%)	1.17 (0.22–6.08)	0.855
**PgR negative**				
Arm B	5 (29.4%)	12 (70.6%)	1	
Arm A	4 (22.2%)	14 (77.8%)	1.46 (0.32–6.70)	0.628
**HR status positive**				
Arm B	8 (44.4%)	10 (55.6%)	1	
Arm A	6 (46.2%)	7 (53.8%)	0.93 (0.22–3.91)	0.925
**HR status negative**				
Arm B	4 (36.4%)	7 (63.6%)	1	
Arm A	4 (25.0%)	12 (75.0%)	1.71 (0.32–9.11)	0.527

OR, odds ratio.

Supplementary Table 8: Association of hormonal receptor status with a pCR in the mITT efficacy population

Sub-population	No pCR *N (%)*	pCR *N (%)*	OR (95% CI)	*P value*
**ER positive**				
Arm B	10 (43.5%)	13 (56.5%)	1	
Arm A	12 (63.2%)	7 (36.8%)	0.45 (0.19–3.50)	0.207
**ER negative**				
Arm B	8 (44.4%)	10 (55.6%)		
Arm A	7 (36.8%)	12 (63.2%)	1.37 (0.38–5.12)	0.638
**PgR positive**				
Arm B	9 (60.0%)	6 (40.0%)	1	
Arm A	11 (68.8%)	5 (31.2%)	0.68 (0.16–2.99)	0.612
**PgR negative**				
Arm B	9 (34.6%)	17 (65.4%)	1	
Arm A	8 (36.4%)	14 (63.6%)	0.93 (0.28–3.03)	0.900
**HR status positive**				
Arm B	11 (45.8%)	13 (54.2%)	1	
Arm A	12 (63.2%)	7 (36.4%)	0.49 (0.14–1.69)	0.261
**HR status negative**				
Arm B	7 (41.2%)	10 (58.8%)	1	
Arm A	7 (36.8%)	12 (63.2%)	1.20 (0.31–4.59)	0.790

OR, odds ratio.

Supplementary Table 9: Other adverse events reported as possibly, probably, or definitely related to treatment in the mITT safety population

	Arm A (*N* = 38)								Arm B (*N* = 41)							
	Grade 1		Grade 2		Grade 3		Grade 4		Grade 1		Grade 2		Grade 3		Grade 4	
	N	%	*N*	%	*N*	%	*N*	%	*N*	%	*N*	%	*N*	%	*N*	%
Respiratory infection	0	0.0	0	0.0	0	0.0	0	0.0	0	0.0	1	2.4	0	0.0	0	0.0
Otitis	0	0.0	0	0.0	0	0.0	0	0.0	0	0.0	1	2.4	0	0.0	0	0.0
Conjunctivitis	2	5.3	1	2.6	0	0.0	0	0.0	3	7.3	1	2.4	0	0.0	0	0.0
Adriamycin extravasation	0	0.0	0	0.0	0	0.0	0	0.0	0	0.0	1	2.4	0	0.0	0	0.0
Lymphangitis	0	0.0	0	0.0	0	0.0	0	0.0	0	0.0	1	2.4	0	0.0	0	0.0
Allergic reaction	1	2.6	3	7.9	0	0.0	0	0.0	0	0.0	2	4.9	0	0.0	0	0.0
Dysarthria	1	2.6	0	0.0	0	0.0	0	0.0	0	0.0	0	0.0	0	0.0	0	0.0
Folliculitis	1	2.6	0	0.0	0	0.0	0	0.0	0	0.0	0	0.0	0	0.0	0	0.0
Cellulitis	0	0.0	1	2.6	0	0.0	0	0.0	0	0.0	0	0.0	0	0.0	0	0.0
Oral thrush	0	0.0	0	0.0	0	0.0	0	0.0	1	2.4	0	0.0	0	0.0	0	0.0
Chest pain	0	0.0	0	0.0	0	0.0	0	0.0	0	0.0	1	2.4	0	0.0	0	0.0
Hot flushes	0	0.0	0	0.0	0	0.0	0	0.0	1	2.4	0	0.0	0	0.0	0	0.0
Abdominal pain	2	5.3	0	0.0	0	0.0	0	0.0	1	2.4	0	0.0	0	0.0	0	0.0
Bone pain	2	5.3	0	0.0	0	0.0	0	0.0	1	2.4	0	0.0	0	0.0	0	0.0
Decreased visual acuity	1	2.6	0	0.0	0	0.0	0	0.0	1	2.4	0	0.0	0	0.0	0	0.0
Elevated creatinine	1	2.6	0	0.0	0	0.0	0	0.0	0	0.0	0	0.0	0	0.0	0	0.0
Cold sore	0	0.0	0	0.0	0	0.0	0	0.0	1	2.4	0	0.0	0	0.0	0	0.0
Dry mouth	1	2.6	0	0.0	0	0.0	0	0.0	0	0.0	0	0.0	0	0.0	0	0.0
Tearfulness	1	2.6	0	0.0	0	0.0	0	0.0	3	7.3	0	0.0	0	0.0	0	0.0
Labial herpes	1	2.6	0	0.0	0	0.0	0	0.0	0	0.0	0	0.0	0	0.0	0	0.0
Tremors	0	0.0	0	0.0	0	0.0	0	0.0	1	2.4	0	0.0	0	0.0	0	0.0
Phlebitis	0	0.0	0	0.0	0	0.0	0	0.0	2	4.9	1	2.4	0	0.0	0	0.0
Nasal congestion	1	2.6	0	0.0	0	0.0	0	0.0	0	0.0	0	0.0	0	0.0	0	0.0
Glassy-eyed appearance	1	2.6	0	0.0	0	0.0	0	0.0	0	0.0	0	0.0	0	0.0	0	0.0
Cough	1	2.6	0	0.0	0	0.0	0	0.0	0	0.0	0	0.0	0	0.0	0	0.0
Hyperphosphatemia	1	2.6	0	0.0	0	0.0	0	0.0	0	0.0	0	0.0	0	0.0	0	0.0
Micturition pain	1	2.6	0	0.0	0	0.0	0	0.0	0	0.0	0	0.0	0	0.0	0	0.0
Loss of consciousness	0	0.0	0	0.0	0	0.0	0	0.0	1	2.4	0	0.0	0	0.0	0	0.0
Cold	0	0.0	0	0.0	0	0.0	0	0.0	1	2.4	0	0.0	0	0.0	0	0.0
Candidiasis	1	2.6	0	0.0	0	0.0	0	0.0	1	2.4	0	0.0	0	0.0	0	0.0
Urinary infection	0	0.0	1	2.6	0	0.0	0	0.0	2	4.9	0	0.0	0	0.0	0	0.0
Diminished renal function	0	0.0	0	0.0	0	0.0	0	0.0	1	2.4	0	0.0	0	0.0	0	0.0
Rhinorrhea	0	0.0	0	0,0	0	0,0	0	0,0	1	2,4	0	0,0	0	0,0	0	0,0
High platelet number	0	0.0	2	5.3	0	0.0	0	0.0	0	0.0	0	0.0	0	0.0	0	0.0
Onicopathy	1	2.6	0	0.0	0	0.0	0	0.0	1	2.4	1	2.4	0	0.0	0	0.0
Blurry vision	2	5.3	0	0.0	0	0.0	0	0.0	0	0.0	0	0.0	0	0.0	0	0.0
Cramping	1	2.6	0	0,0	0	0,0	0	0,0	0	0,0	0	0.0	0	0,0	0	0,0
Extremity pain	1	2.6	1	2.6	0	0.0	0	0.0	0	0.0	0	0.0	0	0.0	0	0.0
Photophobia	1	2.6	0	0.0	0	0.0	0	0.0	0	0.0	0	0.0	0	0.0	0	0.0
Insomnia	3	7.9	0	0.0	0	0.0	0	0.0	2	4.9	0	0.0	0	0.0	0	0.0
Nervousness	0	0.0	0	0.0	0	0.0	0	0.0	1	2.4	0	0.0	0	0.0	0	0.0
Gonalgia	0	0.0	0	0.0	0	0.0	0	0.0	1	2.4	0	0.0	0	0.0	0	0.0
Gingivitis	0	0.0	0	0.0	0	0.0	0	0.0	1	2.4	0	0.0	0	0.0	0	0.0
Metrorrhagia	0	0.0	0	0.0	0	0.0	0	0.0	1	2.4	0	0.0	0	0.0	0	0.0
Xerostomia	0	0.0	0	0.0	0	0.0	0	0.0	1	2.4	0	0.0	0	0.0	0	0.0
Acute gastroenteritis	0	0.0	0	0.0	0	0.0	0	0.0	1	2.4	0	0.0	0	0.0	0	0.0
Pain	0	0.0	0	0.0	0	0.0	0	0.0	2	4.9	0	0.0	0	0.0	0	0.0
Fluid retention	0	0.0	0	0.0	0	0.0	0	0.0	1	2.4	0	0.0	0	0.0	0	0.0
Hyperpigmentation	0	0.0	0	0.0	0	0.0	0	0.0	1	2.4	0	0.0	0	0.0	0	0.0
Hand-Foot syndrome	0	0.0	0	0.0	0	0.0	0	0.0	1	2.4	0	0.0	0	0.0	0	0.0
Arthrosis	0	0.0	0	0.0	0	0.0	0	0.0	1	2.4	0	0.0	0	0.0	0	0.0
Polyphagia	1	2.6	0	0.0	0	0.0	0	0.0	0	0.0	0	0.0	0	0.0	0	0.0
**Total**	30		9		0		0		37		10		0		0	

Supplementary Table 10: Description of the serious adverse events (SAEs) reported

Patient code	Treatment Arm	SAE	Related to treatment
624	A	Respiratory infection	Unrelated (Influenza Type A infection)
1210	A	Febrile neutropenia (grade 4)	Possible (FEC-related)
1510	A	Septic arthritis	Possible (Neoadjuvant treatment-related)
1503	B	Febrile neutropenia (grade 3)	Definite (FEC-related)
1903	B	Febrile neutropenia (grade 3)	Definite (FEC-related)

Supplementary Table 11: Descriptive of LVEF changes in the mITT population

	Arm A	Arm B		
**Baseline**	*N* = 38	*N* = 41		
Mean ± SD	64.38 ± 6.54	64.55 ± 5.68		
Median (P25, P75)	65.00 (58.00, 69.25)	64.00 (61.00, 68.50)		
**Week 12**	*N* = 34	*N* = 40		
Mean ± SD	62.10 ± 6.73	63.09 ± 6.96		
Median (P25, P75)	61.50 (56.75, 66.75)	62.50 (58.00, 68.00)		
**End of treatment**	*N* = 32	*N* = 37		
Mean ± SD	59.67 ± 5.07	60.04 ± 6.41		
Median (P25, P75)	61.00 (55.25, 63.50)	58.00 (56.00, 64.50)		
**Change (End-Baseline)**	*N* = 32	*N* = 37	**Mean difference (95% CI)**	***P* value**
Mean ± SD	–3.96 ± 6.27	–4.68 ± 5.89	0.72 [–2.21 to 3.64]	0.625
Median (P25, P75)	–4.00 (–6.00, –1.78)	–5.00 (–7.50, –1.00)	0.00 [–2.00 to 3.00]	0.754
